# Do Danish medical students feel prepared to deliver healthcare to patients with backgrounds different from their own? A cross-sectional survey

**DOI:** 10.1186/s12909-024-06371-5

**Published:** 2024-11-26

**Authors:** J. Sorensen, A.L. Hindhede, J.S. Ohlendorff, C.J. de Montgomery, S. Maheswaran, M. Norredam, A. Krasnik

**Affiliations:** 1 Department of Public Health, University of Copenhagen, Oester Farimagsgade 5A, Copenhagen, DK- 1353 Denmark; 2 Center for Health Research, Rigshospitalet, Ryesgade 27, Copenhagen, DK-2200 Denmark

**Keywords:** Medical students, Junior physicians, Medical education, Diversity, Preparedness, Helplessness

## Abstract

**Background:**

Migrant and ethnic minority populations in Europe are growing, leading to a demand for health systems and health educations to accommodate the growing diversity. Research indicates that health professionals feel inadequately prepared to care for diverse populations, and medical education has gaps in addressing these issues. The aim of the study is to explore whether Danish medical students and newly graduated physicians feel prepared to meet the needs of the increasingly diverse populations.

**Methods:**

An online survey was emailed to students in the four medical Master’s programs in Denmark and to newly graduated physicians in clinical internships. Data was collected spring 2023. Descriptive statistics reported overall numbers, and multivariate logistic regression was used to model the association between survey answers and the background variables: semester, gender/sex, parent’s education, self-identified ethnicity.

**Results:**

Many medical students and newly graduated physicians feel unprepared to care for patients of backgrounds different from their own. For all items women reported feeling more unprepared than men. Among the medical students and newly graduated physicians with enough clinical experience to answer the question, 34.2% reported feeling helpless at times or often in the past year when treating culturally different patients. Men were less likely than women to report feeling helpless (OR = 0.42; 95% CI 0.28 to 0.64); and self-identified ethnic minorities were 2.59 times more likely than Danish/European medical students and newly graduated physicians to report feeling helpless (95% CI 1.32–5.07).

**Conclusion:**

Our findings indicate shortcomings in medical students and newly graduated physicians feeling of preparedness to provide care to patients of backgrounds different from their own. These findings can assist medical education management with identifying curriculum gaps.

**Supplementary Information:**

The online version contains supplementary material available at 10.1186/s12909-024-06371-5.

## Background

Medical education undergoes constant transformation due to technological and scientific advances, digitalization, and societal needs [1]. The consequent demands on future health professionals’ competencies are many, complex and ever-changing. The growing diversity within the population, caused for instance by increasing migration, may further add to the complexity of the competencies required to effectively address patients’ healthcare needs [2]. Training medical students in diversity competences [3] has been proposed to enhance their self-perceived preparedness to provide care for ethnic minorities [4–6]. However, although medical programs in Denmark and Europe have begun implementing diversity competence training into the curriculum, recent studies suggest that there are still gaps related to this topic [5, 6]. Studies exploring medical students’ opinions as to their levels of preparedness about providing treatment to patients with backgrounds different from their own, reveal a consistent trend of inadequate preparedness [7–9]. However, a study from 2011 among recently graduated medical doctors from Aarhus University found that 83% of the respondents generally felt well-prepared in relation to core learning outcomes like doing a physical examination or taking patients history [10]. Incorporating diversity competence into curricula has posed challenges due to unclear learning content, scheduling, and organization; inadequate support from management, including in resource allocation; and a shortage of qualified educators proficient in this area, as well as a lack of evaluation of the impact of educational initiatives [11]. If medical doctors are inadequately prepared to deliver services to certain segments of the population they serve, it could adversely impact the quality of care provided and, ultimately, contribute to health inequities. During the last two decades, research has documented significant health disparities between migrants and ethnic minorities and the majority population [12]. Among various factors, such disparities may be attributed to barriers—both formal (e.g. legal restrictions and organizational structures) and informal (characteristics of patients and health professionals, e.g. competences, language, health literacy)—faced by migrants and ethnic minorities when trying to access health services [13].

The transition from being a student to becoming a professional physician presents significant challenges, and medical students frequently report feeling anxious and unprepared to make this transition [14, 15]. A recent study from Aggarwal et al. (2023) tries to conceptualize “preparedness for practice” and suggests that “preparedness” combines the constructs of competence, self-confidence (self-efficacy, self-concept), capability and adaptability [16]. Huray et al. (2023) attempt to define “readiness to practice” through content analyses and propose that being “work-ready” requires skills and competencies from professional development activities and placement experience, within communication, self-perceived confidence, clinical skills, and integration of knowledge into practice [17]. Conversely, a concept closely related to the absence of feeling prepared, and common in the transition from student to physician, is “helplessness,” which is defined as the feeling or state of being unable to do anything to help yourself or anyone else [18]. Based on these related concepts of preparedness and helplessness, the aim of this study is to investigate whether Danish medical students and newly graduated physicians feel prepared to deliver healthcare to patients with backgrounds different from their own. We further explored whether the feeling of preparedness varies based on personal characteristics such as gender and ethnicity. Denmark was an ethnically relatively homogeneous country until the 1960s when international migration accelerated, primarily driven by economic factors, as labor migrants contributed to the country’s economic growth and industrial expansion [19]. Aside from a brief immigration halt in the wake of the oil crisis at the beginning of the 1970s, there has been a continuous influx of migrants coming to Denmark, contributing to the diversification of the population. Although the topic of migration and diversity has been a focus in public debate for several decades, the topic of diversity competence has not been given much attention in the discussion of inequities in health and has not been prioritized in medical education [5].

## Methods

### Survey

We gathered information about attitudes, experiences and self-perceived competencies through a survey circulated to all medical schools in Denmark. The survey consisted of 74 items, of which four items were open-ended and thirteen items were about the students’ socio-demographic background. The questionnaire was divided into two parts investigating (1) preparedness and attitudes towards diversity and cross-cultural care (32 items) and part (2) Discrimination experienced during medical school (29 items). The items in part 1 about preparedness came from Sects. 4 and 6 of the questionnaire “Residency Training in Cross-Cultural Care” [20] for which permission to use was obtained. The questionnaire has been validated and has been used in several studies across countries, which provides good possibilities to make comparisons. The items about attitudes in part 1 come from the validated questionnaire “Cross-Cultural Competence instrument for Healthcare Professionals” [21]. The items in part 2 were inspired by the Everyday discrimination scale [22]. This article presents the data from part 1 about students’ preparedness to deliver healthcare services to patients with backgrounds different from their own, and their feelings of helplessness in such encounters (9 items). See Appendix A for full questionnaire in Danish as well as a version in English.

The survey used a 5-point Likert scale for the items related to preparedness, with the following response options: very unprepared, somewhat unprepared, somewhat prepared, well-prepared, and very well-prepared. For the question about feeling helpless a 4-point scale was used with the response options: never, rarely, sometimes, often. For this question the students were also provided with the option, “Not enough patient contact” as it assumed clinical experience. The English language parts of the questionnaire underwent forward-backward translation into Danish by professional translators, thereby ensuring that the implied meaning of the content was preserved. Using the draft questionnaire, six medical students and two junior physicians took part in cognitive interviews [23] to catch possible sources of response errors. Participants were instructed to “think out loud” [23]—verbalizing their thoughts as they responded to the questionnaire—for the researchers to gain insight into whether the participants perceived the questions as intended, and what motivated their answers. The participants were also prompted to reflect on queries by the interviewer. The cognitive interviews provided insights on where to adjust the wording of some of the questions or definitions. For instance, we changed the response category from “not relevant for me” to “not enough patient contact.” The introduction to the survey stated the purpose and aim of the study, informing respondents that their participation was voluntary and that they could revoke their consent at any time, and asking for consent by checking a box. The survey was programmed in SurveyXact developed by Rambøll, version 2021.

### The setting

The study was conducted in Denmark among medical students pursuing their master’s degrees within one of the four Danish medical programs and newly graduate physicians taking their Clinical Basic Education (CBE). The Danish medical programs differ in many ways, e.g. number of students, curriculum, methods, geographical placement, etc. After the students receive their Master degree, they enroll in the mandatory Clinical Basic Education, which is the first step in the further medical training that all doctors must complete in order to obtain permission to work independently. The medical program at the University of Copenhagen (UCPH) is the oldest and is based in the capital, which is also the most populated region in Denmark. The program has a yearly intake of 680 students and could be perceived as more traditional in its approaches and views on medicine and medical education. In the Bachelor’s program the courses are primarily theoretical, offering students minimal patient contact, where the Master’s program includes most of the clinical training in hospitals and general practice clinics. Diversity competence is included at a relatively low level in the curriculum at UCPH. Aarhus University (AU), situated in Denmark’s second-largest city, became the second university in Denmark in 1933 to provide medical education. AU has a yearly uptake of 600 medical students, and the Bachelor and Master programs are like the ones of UCPH. The University of Southern Denmark (SDU) has offered medical education since 1966, and teaching and curriculum are inspired by a Problem-Based Learning (PBL) approach. SDU accepts 140 students every year. The newest medical program, established in 2012, is at Aalborg University (AAU), situated in the fourth-largest city and northernmost part of Denmark. The program is a fully PBL curriculum and annually enrolls approximately 180 students. At Aalborg University the students are introduced to clinical work during the Bachelor’s program.

### Recruitment

We included medical students enrolled in one of the four medical programs in Denmark, from the seventh to the twelfth semesters inclusive, and newly educated junior physicians enrolled in Clinical Basic Education. We excluded students from earlier semesters as we wanted the students to have experienced some clinical training and patient encounters. The survey was distributed to the medical students via their student email from the Course administration at the four universities and to the newly educated junior physicians enrolled at the Clinical Basic Education from the Education section at the National Health Authority, who is responsible for the Clinical Basic Education. Approximately one month after the initial email, a reminder was sent. To maximize survey exposure, additional reminders were posted in student Facebook groups and in the Facebook group for junior physicians at intervals of two weeks, one month, and two months following the distribution of the survey. Additionally, cards with QR codes leading directly to the survey were distributed by one or two colleagues at each university. Moreover, some students contributed by posting on Facebook and Instagram, encouraging other students to answer the survey, and thereby increasing awareness among fellow students. In total the survey was distributed to 5,227 medical students and newly educated junior physicians and 645 completed the survey yielding a response rate of 12%. Data was collected from March 1 through July 17, 2023.

### Outcome variables

The outcome variables were the feelings of preparedness to deal with eight different clinical situations and helplessness in one situation among medical students and junior physicians. The response options were dichotomized into “Very unprepared” and “Somewhat unprepared” versus “Somewhat prepared”, “Well-prepared”, “Very well-prepared” for eight questions about perception of preparedness and “Never” and “Rarely” versus “Sometimes” and “Often” for the one question about feeling helpless when caring for patient with a cultural background different from their own.

### Explanatory variables

The explanatory variables were university, semester, gender, parents’ education and self-identified ethnicity. Gender was included as a binary variable (male vs. female), omitting respondents that identified as non-binary, self-identified gender, or other due to too few responses in those categories (*n* < 10). Self-identified ethnicity was grouped into Danish/European and African/Middle Eastern/South American/Asian, as response numbers did not allow grouping into more specific categories. The ones who did not wish to answer the question about ethnicity were removed (*N* = 7). Parents’ education was categorized with seven levels ranging from Elementary School to Other education e.g. PhD, roughly corresponding with ISCED [24]. Semesters were grouped into 7–9 and 10–12 for university students and 13–14 for CBE.

### Statistical procedures

We used descriptive statistics to report on participants’ characteristics and the overall numbers for preparedness and helplessness. A multivariate logistic regression model was used to estimate the association between each outcome variable and the explanatory variables: gender, ethnicity and parents’ education, and an interaction between university and semester. We report odds ratios (with 95% confidence intervals and p-values) for gender and ethnicity. Only complete cases were considered. Additionally, sensitivity analyses were conducted for the university students with no interaction between university and semester, and separately for the CBE students, including only gender, parents’ education, and ethnicity. The statistical analyses were performed with the software R version 4.3.3 for Linux.

### Ethics

The study was reported to the Data protection committee at the University of Copenhagen (514–0814/23-3000). The study was conducted in accordance with general ethical codes within the field of research, cf. the Declaration of Helsinki. Data was handled in full accordance with the requirements defined by the Danish Data Protection Agency. According to the Danish Act on the Biomedical Ethics Committee System and the Processing of Biomedical Research Projects, the study was not notifiable to the Danish Research Ethics Committee System, as it did not include biological material.

### Characteristics of participants

Of the 645 participants in the survey, 86.4% were enrolled in one of the four Danish medical programs, 60.8% were in their 10th to 12th semester, and 13.6% were undertaking their Clinical Basic Education. 70.3% were women and 28.5% men. Almost 90% self-identified as being of Danish or European ethnicity, whereas 9.0% self-identified their ethnicity as African, Middle Eastern, South American, or Asian and 1% preferred not to disclose their ethnicity. See supplementary materials, Appendix B for more background information.

## Results

### Results on preparedness

Table 1 displays medical students’ preparedness to care for patients with various characteristics. 73.2% of the students declared that they were very unprepared or somewhat unprepared to deliver healthcare services to “newly arrived immigrants” and just 7.7% answered that they were somewhat prepared, well-prepared, or very well-prepared. Additionally, 68.8% reported being very unprepared or somewhat unprepared to care for “patients whose religious beliefs affect treatment” and 68.6% expressed being very unprepared or somewhat unprepared to care for “patients with a distrust of the Danish healthcare system. The students felt most prepared to deliver healthcare services to “patients from different cultures” or “members of ethnic minorities.” However, only 60.9% and 62%, respectively, indicated that they felt somewhat prepared, well-prepared, or very well-prepared.

**Table 1:** Medical students’ preparedness (N/%)

How Prepared Do You Feel to Care for Patients…	Very unprepared	Somewhat unprepared	Somewhat prepared	Well-prepared	Very Well-prepared
1.1.11 …of cultures different from your own?	82 (12.7%)	171 (26.5%)	250 (38.8%)	132(20.5%)	10 (1.6%)
1.1.12 …with health beliefs or practices at odds with Western medicine?	193 (29.9%)	210 (32.6%)	162 (25.1%)	72 (11.2%)	8 (1.2%)
1.1.13 …with a distrust of the Danish healthcare system?	219 (34.0%)	223 (34.6%)	136 (21.1%)	58(9.0%)	9 (1.4%)
1.1.14 …with limited Danish proficiency?	119 (18.4%)	196 (30.4%)	230 (35.7%)	86 (13.3%)	14 (2.2%)
1.1.15 …who are newly arrived immigrants?	289 (44.8%)	183 (28.4%)	123 (19.1%)	39 (6.0%)	11 (1.7%)
1.1.16 …whose religious beliefs affect treatment?	231 (35.8%)	213 (33.0%)	137 (21.2%)	51(7.9%)	13 (2.0%)
1.1.17 …who use alternative medicines?	120 (18.6%)	194 (30.1%)	214 (33.2%)	97 (15.0%)	20 (3.1%)
1.1.18 …who are members of ethnic minorities?	77 (11.9%)	168 (26.0%)	218 (33.8%)	147 (22.8%)	35 (5.4%)

Men were less likely than women to express feeling unprepared (OR = 0.26; 95% CI 0.18–0.38) when treating “patients whose religious beliefs affect treatment” (Table 2). When it comes to treating patients who are “newly arrived immigrants,” men were also less likely than women to report feeling unprepared (OR = 0.29; 95% CI 0.20–0.42) and the same was the case regarding “patients with a distrust of the Danish healthcare system” (OR = 0.31; 95% CI 0.21–0.45). In general, for all items about preparedness, men were less likely to report feeling unprepared (Table 2). There was no significant difference in the feeling of preparedness between medical students who self-identified with an ethnicity other than Danish/European and the Danish/European students, except for treating “patients who use alternative medicine.” Here, students who self-identified with another ethnicity were more likely than those identifying as Danish/European to report feeling unprepared (OR = 2.11; 95% CI 1.15–3.86).

**Table 2:** The importance of gender and ethnicity for medical students’ preparedness to care for patients of backgrounds or characteristics different from their own

		Gender		Ethnicity	
How Prepared Do You Feel to Care for Patients.		Women	Men	Danish/ European	African/Middle Eastern/ South American/Asian
1.1.11 … from cultures different from your own?	OR	Ref	0.46	Ref	1.07
	CI 95%	Ref	[0.31;0.68]	Ref	[0.60;1.92]
	p-value	-	< 0.0001	-	0.81
1.1.12 … with health beliefs or practices at odds with Western medicine?	OR	Ref	0.44	Ref	0.88
	CI 95%	Ref	[0.30;0.62]	Ref	[0.49;1.60]
	p-value	-	< 0.0001	-	0.67
1.1.13 … with a distrust of the Danish healthcare system?	OR	Ref	0.31	Ref	1.21
	CI 95%	Ref	[0.21;0.45]	Ref	[0.63;2.35]
	p-value	-	< 0.0001	-	0.57
1.1.14 … with limited Danish language proficiency?	OR	Ref	0.49	Ref	1.09
	CI 95%	Ref	[0.34;0.70]	Ref	[0.62;1.94]
	p-value	-	< 0.0001	-	0.76
1.1.15 … who are newly arrived immigrants?	OR	Ref	0.29	Ref	0.75
	CI 95%	Ref	[0.20;0.42]	Ref	[0.39;1.42]
	p-value	-	< 0.0001	-	0.38
1.1.16 … whose religious beliefs affect treatment?	OR	Ref	0.26	Ref	0.67
	CI 95%	Ref	[0.18;0.38]	Ref	[0.36;1.25]
	p-value	-	< 0.0001	-	0.21
1.1.17… who use alternative medicine?	OR	Ref	0.42	Ref	2.11
	CI 95%	Ref	[0.29;0.61]	Ref	[1.15;3.86]
	p-value	-	< 0.0001	-	0.016
1.1.18 … who are members of ethnic minorities?	OR	Ref	0.39	Ref	1.34
	CI 95%	Ref	[0.26;0.57]	Ref	[0.75;2.40]
	p-value	-	< 0.0001	-	0.32

*Notes* Multiple logistic regression model adjusted for gender, ethnicity and parents’ education, and an interaction between university and semester

### Results on helplessness

Table 3 shows that 34.2% of the students reported that they sometimes or often feel helpless in relation to the treatment of patients of a culture different from their own. Table 4 demonstrates that when it comes to feeling helpless in relation to the treatment of patients of a culture different from their own, men were less likely than women to report feeling helpless (OR = 0.42; 95% CI 0.28 to 0.64). Medical students who self-identify with an ethnicity other than Danish/European were 2.59 times more likely than students with Danish/European ethnicity to report feeling helpless (95% CI 1.32–5.07).

**Table 3:** Medical students’ feeling of helplessness in relation to the treatment of patients with a culture different from one’s own

	Never	Rarely	Sometimes	Often	Not enough patient contact	Total
In the last year, how often have you felt helpless about what to do when providing care involving patients of a culture different from your own?	85 (13.2%)	215 (33.3%)	188 (29.1%)	33 (5.1%)	124 (19.2%)	645 (100%)

**Table 4:** The importance of gender and ethnicity for medical students’ feeling of being helpless in relation to the treatment of patients of a culture different from their own

Variable	Entity	Odds ratio	95% CI	*p*-value
**Gender**	Women	Ref	-	-
	Men	0.42	[0.28;0.64]	< 0.0001
**Ethnicity**	Danish/European	Ref	-	-
	African/Middle Eastern/ South American/Asian	2.59	[1.32;5.07]	0.0056

*Notes* Multiple logistic regression model adjusted for gender, ethnicity and parents’ education, and an interaction between university and semester

### Sensitivity analyses

The sensitivity analyses showed little change in the estimated effect of gender and ethnicity (see Tables 7 and 8 in Appendix C).

## Discussion

Many medical students and newly graduated physicians felt unprepared to care for several categories of patients. This ranged from one third of the students feeling unprepared (the lowest number) to over two thirds (the highest number) across various items. The items for which most medical students reported feeling unprepared were “newly arrived immigrants,” “patients whose religious beliefs affect treatment” and “patients with a distrust of the Danish healthcare system.” Women reported feeling more unprepared than men. Overall, over one third of students and young physicians reported having felt helpless at times, or often, in the past year when treating patients of cultures different from their own. Men reported feeling less helpless in caring for patients from a culture other than their own than did women; and self-identified ethnic minorities reported feeling helpless more often than students identifying as Danish/European.

Caring for newly arrived migrants can confront healthcare providers with challenges not encountered as frequently or in the same way in the majority population. This minority patient group is especially complex, since there are many factors at play when it comes to their interaction with health services. The language barrier is a major issue, and research indicates that medical students lack experience or training in working with interpreters [5], although many studies have highlighted interpretation as a key element in delivering healthcare services to migrants [3, 25]. Another important challenge when working with newly arrived migrants is that they are unfamiliar with the host country’s healthcare system (e.g. lack of knowledge about how to navigate and utilize it [26]). This can lead to incorrect use of the healthcare system, causing frustration among health professionals or lower satisfaction among migrants, as their expectations are not met [27].

Much research has documented differences between genders among physicians and medical students. For instance, studies suggest that women are more empathic than men [28, 29], when measuring empathy using self-reporting tools. Our data also reveal a gap between genders when it comes to feeling prepared to care for patients with a background different from their own. A systematic review from 2020 investigating the confidence gap between male and female physicians [30] indicated that while there were no disparities in clinical performance, women tend to assess shortcomings in their own abilities more frequently than men. This could partly explain why woman in our study report feeling unprepared more often than men.

A study from 2016 investigating the association between gender and attitudes towards doctor-patient communication found that women would describe doctor-patient communication using positive attributes like “openness” and “empathy” whereas men associated this with more neutral attributes as “uptake of medical history” [31]. This suggests that women might place greater importance on doctor-patient communication dynamic. Our questionnaire probes participants about their preparedness to interact and communicate with different patient groups and since women may weigh the importance of this topic more than men, they might also have reflected more about the difficulties and challenges involved in doctor-patient communication. In this way, the gender differences in feeling prepared may have more to do with how conscious women are relative to men about these issues than with any real difference in preparedness to provide healthcare.

A study conducted by Lopez et al. (2008) in the US using the same questions and statistical methods as our study, showed that some physicians’ groups from ethnic minorities reported more preparedness to care for minorities than physicians of the majority ethnicity [9]. Our results (see Table 2) except one, are not consistent with this, since there was no major difference in the feeling of preparedness between students identifying as Danish/European and students identifying as ethnic minorities. This may be explained by a common perception in Denmark of some ethnic minority patients being challenging due to their distrust or newness as immigrants in relation to Danish healthcare, contrary to the more established minority patients in the US. Only regarding the item “patients who use alternative medicines” do our results align with the findings of Lopez et al., who also found that ethnic minority physicians reported feeling less prepared when it comes to “patients who use alternative medicine” than physicians of the majority ethnicity.

The medical programs’ curricula are designed to prepare students for their journey towards becoming physicians. But what exactly does it mean “to be prepared”? According to Aggarwal et al. (2023) preparedness encompasses four factors, with *confidence* being one crucial element [16]. To be confident in something does not necessarily imply *competence*, also one of the four factors identified by Aggerwal. Confidence could partly stem from insufficient insight into concrete challenges in daily clinical work. Our results show that when the medical students are asked in more general terms about treating patients from other backgrounds, they are somewhat more “confident” than when they are asked about more specific challenges connected to certain patient characteristics (e.g. “newness,” or mistrust). Similarly, a recent study posed a specific question to Danish medical students regarding their confidence levels in diagnosing dermatological diseases on dark skin compared to white skin [32]. The findings revealed that they expressed lower confidence in diagnosing conditions on dark skin than on white skin. Furthermore, research has delved into reasons behind students’ sense of unpreparedness in clinical practice, highlighting issues such as information overload, a gap between theoretical knowledge and its application, negative emotions, and the learning process itself [33]. Other studies mention prescribing medicine, time/workload, being part of a team, adjusting to professionals’ roles and hierarchy as reasons why medical students feel unprepared [34, 35]. The factors contributing to a sense of unpreparedness in medical practice are numerous and complex. The lack of “confidence” may simply signify an acknowledgment that as a newly graduated physician, one may not be fully ready for every task or scenario encountered. Instead, it might be seen as an acceptance that the journey towards becoming a professional physician involves continuous evolution and preparedness for ongoing learning.

Our findings, however, indicate a substantial feeling of being unprepared for caring for patients from cultural backgrounds different from their own, and that women and ethnic minorities, who frequently express feeling less prepared, also tend to experience a sense of helplessness more frequently. This feeling is in some studies linked to difficult clinical encounters related to patient, physician or situation, or a combination of all three, and where a disparity of expectations, perceptions or actions exist [36]. Among factors contributing on the physician’s side to these difficult encounters are emotional burnout, insecurity, exhaustion, and anxiety [36]. Many of these factors are reported more often by woman than men [14, 15] and could be a contributing factor to why women are more inclined to report feeling helpless compared to men. A possible contributing factor regarding the feeling of helplessness in Danish ethnic minority medical students, could be their direct experience of belonging to a minority group within the Danish healthcare system, giving them insight into the associated challenges. They may have encountered difficult encounters between physicians and patients firsthand or through their relatives, which could lead to a greater tendency to report feelings of helplessness. This could be caused by heightened awareness stemming from their personal experiences and recognition of their own role in such encounters.

### Strengths and limitations

The study has several limitations, with the most obvious being low response rate. Other studies conducted among medical students in Denmark using different methods, including direct emails to the students’ personal electronic mailboxes and social media recruitment, also yielded relatively low response rates [29, 32]. Despite our approach with emails sent to the students’ university email addresses along with reminders on social media, some students stated they did not use university email frequently due to bombardment of notifications, causing them only to check necessary emails, such as for examination sign-ups. Given that young people’s communication preferences involve social media platforms, reaching medical students via email seems a challenge. Another explanation is that students are overburdened with study and clinical work. Another point is that although we tried to keep the questionnaire short, the low response rate could indicate that it was too long or could not capture the students’ interest. Secondly, there is a significant risk of selection bias as the low response rate may indicate that respondents had a specific interest in the subject matter. When it comes to gender and self-identified ethnicity, the sample seems representative compared with data about the entire UCPH medical student population. Rather than focusing solely on precise numbers, the emphasis should be on the overall trend of the study as the primary take-home message. This can be reinforced in further studies by employing additional methods such as reviewing curricula, conducting individual interviews, and organizing focus group discussions. Thirdly there could be a risk of recall bias since the students are asked to evaluate experiences over a longer time span and therefore may not remember some relevant study experiences. One strength of the study is the inclusion of all medical programs in Denmark and despite the low response rate, this resulted in a relatively high number of responses. Furthermore, the use of validated and tested questionnaire questions adds strength to the study, given their extensive utilization in data collection and research [7–9, 20].

## Conclusion

The results of this study can assist medical education management in identifying curriculum gaps and shortcomings. For instance, communication courses could address difficult encounters and how to handle patients with characteristics such as being new immigrants, being mistrustful or utilizing alternative medicine. Further research of qualitative nature into what it means to “feel prepared,” and of what role gender and ethnicity play in the feeling of “being prepared” is needed to unfold and understand the underlying dynamics and qualify the curriculum further.

## Electronic supplementary material

Below is the link to the electronic supplementary material.

Supplementary Material 1

Supplementary Material 2

Supplementary Material 3

## Data Availability

Data can be retrieved from the first author upon reasonable request.
