# Supplementary material for: Do Danish medical students feel prepared to deliver healthcare to patients with backgrounds different from their own? A cross-sectional survey
Source: BMC Med Educ. 2024 Nov 26;24:1366. doi: 10.1186/s12909-024-06371-5 (PMC11590545; doi:10.1186/s12909-024-06371-5)
Supplement: Supplementary file 1 — Supplementary Material 1 [file 12909_2024_6371_MOESM1_ESM.pdf]

The English version is a translation from Danish; however, questions 1.1.1 to 1.1.19 have not been translated, as they were originally in English

Competencies for treating patients with diverse backgrounds and discrimination of medical students

Thank you very much for taking the time to answer our questionnaire.

When you answer our questions, your responses help us 1) work to improve the quality of the content in medical education so that future doctors' competencies match patient needs, and 2) improve the study of environment for students who experience discrimination.

The questionnaire consists of two parts, which are independent of each other – they are two separate surveys, each with its own focus.

Part one – aims to investigate your knowledge and attitudes towards providing treatment to patients with diverse backgrounds.

Part two – aims to investigate whether you have experienced discrimination based on your sexual orientation, religion, gender, ethnicity, or disability during your medical education.

In addition to the questions in part one and two, you will be asked to answer some questions about yourself and your background.

It takes approx. 15 minutes to complete the questionnaire. Please note that there are no right or wrong answers.

It is not possible to save your answers and return to the questionnaire later – it must be completed in one sitting. Note that the questionnaire has not been submitted until you have clicked "**Finish**".

If you have any questions regarding the questionnaire, please contact Janne Sørensen, jans@sund.ku.dk or by phone at 35327288.

Distribution and security

All data is processed anonymously and in accordance with the General Data Protection Regulation (EU GDPR). The results will be reported in a combined form, so that the individual responses cannot be identified.

Consent

I hereby consent to the information that I provide in the questionnaire to being used for Dissemination and publication. ☐

1 Knowledge and attitudes towards providing treatment to patients with diverse backgrounds

In part one, there are a number of questions focusing on your knowledge or and attitudes towards providing treatment to patients with diverse backgrounds. In this questionnaire we define culturally diverse patients as patients who belong to cultures different from your own.

1.1 Competencies acquired during medical education

The questions in this section focus on the competencies and knowledge you have acquired during your medical education in relation to providing treatment to patients with diverse backgrounds and how prepared you feel to interact with them.

**How much of the medical school curriculum has been spent on cross-cultural aspects, in relation to…**	None	Very little	Some	A lot
1.1.1 Determining how a patient (or the patient's family) wants to be addressed and interacted with?	☐	☐	☐	☐
1.1.2 Taking a social history?	☐	☐	☐	☐
1.1.3 Assessing the patient's (or the patient's family's) understanding of the cause of their illness?	☐	☐	☐	☐
1.1.4 Identifying whether a patient (or the patient's family) is mistrustful of the health care system or the physician?	☐	☐	☐	☐
1.1.5 Negotiating with the patient (or the patient's family) about key aspects of the treatment plan?	☐	☐	☐	☐
1.1.6 Identifying how well a patient (or the patient's family) can read or write Danish?	☐	☐	☐	☐
1.1.7 Identifying religious beliefs that might affect clinical care?	☐	☐	☐	☐
1.1.8 Identifying cultural (non-religious) customs that might affect clinical care?	☐	☐	☐	☐
1.1.9 Identifying how a patient (or the patient's family) makes decisions with other family members?	☐	☐	☐	☐
1.1.10 delivering services effectively through a medical interpreter?	☐	☐	☐	☐

**How prepared do you feel to take care of patients (or patients' families)...**	Very unprepared	Somewhat unprepared	Somewhat prepared	Well-prepared	Very well-prepared	Not relevant for me
1.1.11 from cultures different from your own?	☐	☐	☐	☐	☐	☐
1.1.12 with health beliefs or practices at odds with Western medicine?	☐	☐	☐	☐	☐	☐
1.1.13 with a distrust of the Danish health care system?	☐	☐	☐	☐	☐	☐
1.1.14 with limited Danish proficiency?	☐	☐	☐	☐	☐	☐
1.1.15 who are new immigrants?	☐	☐	☐	☐	☐	☐
1.1.16 whose religious beliefs affect treatment?	☐	☐	☐	☐	☐	☐
1.1.17 who use alternative or complementary medicine?	☐	☐	☐	☐	☐	☐
1.1.18 who are members of ethnic minority groups?	☐	☐	☐	☐	☐	☐

	Never	Rarely	Sometimes	Often	Not relevant for me
1.1.19 In the last year, how often have you felt helpless about what to do when providing care involving patients of a culture different from your own?	☐	☐	☐	☐	☐

1.2 Attitudes toward diversity

In this section, we ask you to consider a series of statements focusing on the interaction between physicians and patients with diverse backgrounds, as well as diversity in society in general.

	completely agree	mostly agree	neither agree nor disagree	mostly disagree	completely disagree	no answer possible
1.2.1 I consider working in a cross-cultural team an enrichment.	☐	☐	☐	☐	☐	☐
1.2.2 I find it an imposition, when people who migrated to Denmark a long time ago, cannot speak Danish properly.	☐	☐	☐	☐	☐	☐
1.2.3 By communicating with patients with a migration background I can learn about different cultural orientations.	☐	☐	☐	☐	☐	☐
1.2.4 Cultural diversity is also an enrichment.	☐	☐	☐	☐	☐	☐
1.2.5 I enjoy talking to people who have migrated to Denmark about their experiences here.	☐	☐	☐	☐	☐	☐
1.2.6 I have the impression that migrants often assume discrimination, when in fact general rules are simply being enforced.	☐	☐	☐	☐	☐	☐
1.2.7 The interaction with people from other cultural backgrounds helps me reflect upon my own cultural background.	☐	☐	☐	☐	☐	☐
1.2.8 I would like to make use of training, advising and educational offers, in order to improve my understanding of patients with a migration background.	☐	☐	☐	☐	☐	☐
1.2.9 I consider it an enrichment to have friendships with people from different cultural backgrounds.	☐	☐	☐	☐	☐	☐

1.2.10 People who migrate to Denmark should adapt to society, not the other way around.	☐	☐	☐	☐	☐	☐
1.2.11 Institutions and the public pay too much attention to the special wishes of migrants.	☐	☐	☐	☐	☐	☐
1.2.12 Institutions and the public are too attentive to immigrants' special desires.	☐	☐	☐	☐	☐	☐
1.2.13 I find it exciting to treat patients with a migration background.	☐	☐	☐	☐	☐	☐

2 Discrimination

In this part of the survey, the focus is on whether you, during your medical education, have experienced discrimination based on your sexual orientation, religion, gender, ethnicity, age, or disability.

2.1 Experiences with discrimination during medical school

Indicate whether you have experienced any of the following types of discrimination. Please note that it should have occurred during your medical education – during clinical placements and/or at the university.

**During medical school, I experienced…**	At least once a week	A few times a month	A few times a year	It has happened once	never
2.1.1 being ignored	☐	☐	☐	☐	☐
2.1.2 not being treated with respect	☐	☐	☐	☐	☐
2.1.3 being ridiculed	☐	☐	☐	☐	☐
2.1.4 not receiving the help I was entitled to	☐	☐	☐	☐	☐
2.1.5 encountering stereotypical remarks	☐	☐	☐	☐	☐
2.1.6 receiving condescending comments	☐	☐	☐	☐	☐
2.1.7 receiving unwelcome comments about clothing/appearance	☐	☐	☐	☐	☐
2.1.8 being told offensive jokes	☐	☐	☐	☐	☐
2.1.9 Other mistreatment (describe in the text field below					

Please describe here any other mistreatment you have experienced:

2.2 Who has subjected you to discrimination?

	Tick one or more boxes
2.2.1 Other students	☐
2.2.2 Teacher/instructor	☐
2.2.3 Medical doctor	☐
2.2.4 Nurse or other healthcare personnel	☐
2.2.5 Healthcare sector management	☐
2.2.6 Administrative staff at the healthcare sector	☐
2.2.7 Administrative staff at the university	☐
2.2.8 Patients	☐
2.2.9 Relatives to patients	☐
2.2.10 Others:_____________________	

2.3 What do you think is the reason behind your experiences?

	Tick one or more boxes
2.3.1 My gender or gender identity	☐
2.3.2 My Ethnicity	☐
2.3.3 My Skin color	☐
2.3.4 My Language	☐
2.3.5 My Sexual orientation	☐
2.3.6 My Religion	☐
2.3.7 My Age	☐
2.3.8 My disability	☐
2.3.9 Other:______________________	

Do you have anything to add, or would you like to describe your experiences? If so, you can do it here:

3 Questions about your background

3.1 Which university or Clinical Basic Education (KBU) are you enrolled in?

Clinical basis education (KBU) ☐

Aalborg University ☐

Aarhus University ☐

University of Copenhagen ☐

University of Southern Denmark ☐

3.2 Which semester are you currently on?

7th semester ☐

8th semester ☐

9th semester ☐

10th semester ☐

11th semester ☐

12th semester ☐

3.3 Which gender do you identify as?

Woman ☐

Man ☐

Non-binary ☐

Prefer not to disclose ☐

I prefer to describe it myself ______________________

3.4 Which sexual orientation best represents you?

- Heterosexual ☐
- Homosexual man ☐
- Homosexual woman ☐
- Bisexual ☐
- Prefer not to disclose ☐
- I prefer to describe it myself ____________________

3.5 Which year where you born?

____

3.6 In which country where you born?

____

3.7 Do you consider yourself to be a part of an ethnic minority in the country where you reside in?

- Yes ☐
- No ☐

3.7a please describe your ethnicity: e.g. Danish –Turkish, German, Danish – Palestinian, Somali, Greenlandic

3.8 In which country were your parents born?

Parent 1. ____________________________
Parent 2. ____________________________

3.9 What is the highest level of education completed by one of your parents?

- Elementary school up to 9 th or 10th grade ☐
- One or more shorter courses (e.g. specialist worker courses, labor market courses, etc.) ☐
- Secondary education (e.g. high school diploma, HF, HHX, HTX) ☐
- Vocational education/trade worked (e.g. office or sales assistant, hairdresser, bricklayer, medical secretary, social and healthcare assistant, farmer) ☐
- Short-cycle higher education, 2-3 years (e.g. marketing economist, police officer, laboratory technician, mechanical technician, data technician, multimedia designer, economist, dental hygienist) ☐
- Medium-cycle higher education, 3-4 years (e.g. primary and lower secondary school teacher, social worker, building constructor, nurse, physiotherapist, Bachelor of Engineering, educationist, bachelor) ☐
- Long-cycle higher education, more than 4 years (e.g. Graduate engineer, Master of arts, medical doctor, Psychologist) ☐

Other education: ______________________________

3.10 Do you have a disability?

Yes
No

3.10a Describe your disability:

Can we contact you for a follow-up qualitative study? If yes, please write your email here:

Remember to click “finish”.

Thank you for your response!

Kompetencer til behandling af patienter med mangfoldige baggrunde og diskrimination af lægestuderende

Tusind tak, fordi du vil tage dig tid til at besvare vores spørgeskema

Når du svarer på vores spørgsmål, hjælper dine svar os med 1) at arbejde for at forbedre kvaliteten af indholdet i undervisningen på lægeuddannelserne, så kommende lægers kompetencer matcher patienternes behov og 2) med at forbedre studiemiljøet for studerende, der oplever diskrimination.

Spørgeskemaet består af to separate undersøgelser med hvert sit fokus.

Del 1 har til formål at undersøge din viden om og dine holdninger til at yde behandling til patienter med mangfoldige baggrunde.
Del 2 har til formål at undersøge, hvorvidt du under din uddannelse til læge har oplevet diskrimination på baggrund af din seksuelle orientering, religion, køn, etnicitet, alder eller funktionsnedsættelse.

Ud over spørgsmålene i del 1 og 2, vil du til sidst blive bedt om at svare på nogle spørgsmål om dig selv og din baggrund.

Det tager ca. **15 minutter** at udfylde spørgeskemaet. Du skal vide, at der ikke er rigtige eller forkerte svar.

Det er ikke er muligt at gemme indtastede svar og forlade spørgeskemaet – det skal udfyldes på en gang. Bemærk, at spørgeskemaet først er indsendt når du har trykket afslut.

Hvis du har spørgsmål vedr. spørgeskemaet, kan du kontakte Janne Sørensen på, jans@sund.ku.dk eller på tlf. 35327288.

Distribution og sikkerhed

Alle data behandles anonymt og i henhold til den generelle databeskyttelsesforordning (EU GDPR). Resultaterne vil blive rapporteret i samlet form, således at de individuelle besvarelser ikke kan identificeres.

❑

Jeg giver hermed samtykke til at de oplysninger, som jeg opgiver i spørgeskemaet, må benyttes i anonymiseret form til formidling og publicering.

1. Viden og holdninger til at yde behandling til patienter med mangfoldige baggrunde.

I del 1 stilles der en række spørgsmål med fokus på din viden om og dine holdninger til at yde behandling til patienter med mangfoldige baggrunde.

I dette spørgeskema definerer vi kulturelt mangfoldige patienter som patienter, som tilhører kulturer, der er anderledes end din egen.

1.1 Kompetencer erhvervet under uddannelsen til læge

Spørgsmålene i dette afsnit fokuserer på, hvilke kompetencer og viden du har erhvervet dig under din uddannelse til læge i relation til at yde behandling til patienter med mangfoldige baggrunde samt hvor forberedt du føler dig til at interagere med dem.

	Intet	Lidt	Noget	Meget
1.1.1 *Hvor meget af undervisningen på medicinstudiet har været brugt på tværkulturelle aspekter, i relation til at...*<br>klarlægge, hvordan en patient (eller patientens familie) ønsker at blive tiltalt og interageret med?	❑	❑	❑	❑
1.1.2 *Hvor meget af undervisningen på medicinstudiet har været brugt på tværkulturelle aspekter, i relation til at...*<br>spørge ind til patientens sociale baggrund?	❑	❑	❑	❑
1.1.3 *Hvor meget af undervisningen på medicinstudiet har været brugt på tværkulturelle aspekter, i relation til at...*	❑	❑	❑	❑

vurdere patientens (eller patientens families) forståelse af årsagen til den pågældendes sygdom?

1.1.4 *Hvor meget af undervisningen på medicinstudiet har været brugt på tværkulturelle aspekter, i relation til at...*

finde ud af, om en patient (eller patientens familie) er mistroisk over for sundhedsvæsenet eller lægen?

❑ ❑ ❑ ❑

1.1.5 *Hvor meget af undervisningen på medicinstudiet har været brugt på tværkulturelle aspekter, i relation til at...*

diskutere vigtige aspekter i behandlingsplanen med patienten (eller patientens familie)?

❑ ❑ ❑ ❑

1.1.6 *Hvor meget af undervisningen på medicinstudiet har været brugt på tværkulturelle aspekter, i relation til at...*

finde ud af, hvor godt en patient (eller patientens familie) læser eller skriver dansk?

❑ ❑ ❑ ❑

1.1.7 *Hvor meget af undervisningen på medicinstudiet har været brugt på tværkulturelle aspekter, i relation til at...*

identificere religiøs tro, der kan påvirke den kliniske behandling?

❑ ❑ ❑ ❑

1.1.8 *Hvor meget af undervisningen på medicinstudiet har været brugt på tværkulturelle aspekter, i relation til at...*

identificere kulturelle (ikke-religiøse) skikke, der kan påvirke den kliniske behandling?

❑ ❑ ❑ ❑

1.1.9 *Hvor meget af undervisningen på medicinstudiet har været brugt på tværkulturelle aspekter, i relation til at...*

finde frem til, hvordan en patient (eller patientens familie) træffer beslutninger sammen med andre familiemedlemmer?

❑ ❑ ❑ ❑

1.1.10 *Hvor meget af undervisningen på medicinstudiet har været brugt på tværkulturelle aspekter, i relation til at...*

levere pleje og behandling på en effektiv måde gennem en medicinsk tolk?

❑ ❑ ❑ ❑

1.1 Kompetencer erhvervet under uddannelsen til læge

	Meget uforberedt	Lidt uforberedt	Lidt forberedt	Velforberedt	Meget velforberedt
1.1.11 *Hvor velforberedt føler du dig til at tage dig af patienter (eller patienters familier)*	❑	❑	❑	❑	❑

...fra kulturer, der er anderledes end din egen?

1.1.12 *Hvor velforberedt føler du dig til at tage dig af patienter (eller patienters familier)*

...med sundhedsmæssige holdninger eller skikke, der ikke stemmer overens med vestlig medicin?

❑ ❑ ❑ ❑ ❑

1.1.13 *Hvor velforberedt føler du dig til at tage dig af patienter (eller patienters familier)*

...som er mistroisk over for det danske sundhedsvæsen?

❑ ❑ ❑ ❑ ❑

1.1.14 *Hvor velforberedt føler du dig til at tage dig af patienter (eller patienters familier)*

...med begrænsede færdigheder i dansk?

❑ ❑ ❑ ❑ ❑

1.1.15 *Hvor velforberedt føler du dig til at tage dig af patienter (eller patienters familier)*

...som er nyankommne indvandrere?

❑ ❑ ❑ ❑ ❑

1.1.16 *Hvor velforberedt føler du dig til at tage dig af patienter (eller patienters familier)*

...hvis religiøse tro påvirker behandlingen?

❑ ❑ ❑ ❑ ❑

1.1.17 *Hvor velforberedt føler du dig til at tage dig af patienter (eller patienters familier)*

...som bruger alternativ medicin?

❑ ❑ ❑ ❑ ❑

1.1.18 *Hvor velforberedt føler du dig til at tage dig af patienter (eller patienters familier)*

...som tilhører etniske minoritetsgrupper?

❑ ❑ ❑ ❑ ❑

1.1 Kompetencer erhvervet under uddannelsen til læge

1.1.19 Hvor ofte har du i det sidste år følt dig afmægtig i forhold til, hvad du skal gøre, når du er i kontakt med patienter med en anden kultur end din egen?

Aldrig	Sjældent	Sommetider	Ofte	Har ikke haft nok patient kontakt
❑	❑	❑	❑	❑

1.2 Holdninger til mangfoldighed

I dette afsnit beder vi dig tage stilling til en række udsagn, der fokuserer på interaktionen mellem læge og patienter med mangfoldige baggrunde samt

til mangfoldighed i samfundet generelt.

	Helt enig	Delvist enig	Hverken enig eller uenig	Delvist uenig	Helt uenig
1.2.1 Jeg mener, at det er en berigelse at arbejde i et tværkulturelt team.	❑	❑	❑	❑	❑
1.2.2 Jeg synes, det er urimeligt, at folk, der er indvandret til Danmark for lang tid siden, ikke taler ordentligt dansk.	❑	❑	❑	❑	❑
1.2.3 Når jeg kommunikerer med patienter med indvandrerbaggrund, lærer jeg om forskellige kulturer.	❑	❑	❑	❑	❑
1.2.4 Kulturel mangfoldighed er også en berigelse.	❑	❑	❑	❑	❑
1.2.5 Jeg kan godt lide at tale med folk, der er indvandret til Danmark, om deres oplevelser her.	❑	❑	❑	❑	❑
1.2.6 Det er mit indtryk, at indvandrere ofte går ud fra, at der er tale om diskrimination, selv om det blot er generelle regler, der skal håndhæves.	❑	❑	❑	❑	❑
1.2.7 Interaktionen med folk fra andre kulturelle baggrunde hjælper mig med at reflektere over min egen kulturelle baggrund.	❑	❑	❑	❑	❑
1.2.8 Jeg vil gerne gøre brug af kurser, rådgivning og pædagogiske tilbud, så jeg kan forbedre min forståelse for patienter med indvandrerbaggrund.	❑	❑	❑	❑	❑
1.2.9 Jeg anser det for en berigelse at være venner med folk fra forskellige kulturelle baggrunde.	❑	❑	❑	❑	❑
1.2.10 Folk, der indvandrer til Danmark, skal tilpasse sig samfundet – ikke omvendt.	❑	❑	❑	❑	❑
1.2.11 Det er vigtigt for mig at behandle patienterne i henhold til deres kulturelle behov og individuelle værdier.	❑	❑	❑	❑	❑
1.2.12 Institutioner og offentligheden er for opmærksomme på indvandrernes særlige ønsker.	❑	❑	❑	❑	❑
1.2.13 Jeg synes, det er spændende at behandle patienter med indvandrerbaggrund.	❑	❑	❑	❑	❑

2. Diskrimination

I denne del stilles der spørgsmål med fokus på, hvorvidt du under din uddannelse til læge har oplevet diskrimination på baggrund af din seksuelle orientering, religion, køn, etnicitet, alder eller funktionsnedsættelse.

2.1 Oplevelser med diskrimination under lægeuddannelsen

Angiv, om du har været udsat for en af følgende former for diskrimination. Vær opmærksom, på at det skal være sket under din uddannelse til læge - under klinik ophold og/eller på universitetet.

	Mindst en gang om ugen	Et par gange om måneden	Et par gange om året	Sket én gang	Aldrig

2.1.1 *På lægeuddannelsen har jeg oplevet ...* at jeg blev ignoreret	❑	❑	❑	❑	❑
2.1.2 *På lægeuddannelsen har jeg oplevet ...* at jeg ikke blev mødt med respekt	❑	❑	❑	❑	❑
2.1.3 *På lægeuddannelsen har jeg oplevet ...* at jeg blev latterliggjort	❑	❑	❑	❑	❑
2.1.4 *På lægeuddannelsen har jeg oplevet ...* at jeg ikke fik den hjælp, jeg havde ret til	❑	❑	❑	❑	❑
2.1.5 *På lægeuddannelsen har jeg oplevet ...* at jeg oplevede stereotype udsagn	❑	❑	❑	❑	❑
2.1.6 *På lægeuddannelsen har jeg oplevet ...* at jeg fik nedladende kommentarer	❑	❑	❑	❑	❑
2.1.7 *På lægeuddannelsen har jeg oplevet ...* at jeg fik uvelkomne kommentarer om tøj/udseende	❑	❑	❑	❑	❑
2.1.8 *På lægeuddannelsen har jeg oplevet ...* at jeg blev fortalt krænkende vittigheder	❑	❑	❑	❑	❑
2.1.9 *På lægeuddannelsen har jeg oplevet ...* Anden dårlig behandling (beskriv i nedenstående tekstfelt)	❑	❑	❑	❑	❑

Beskriv her hvilken anden dårlig behandling, som du har været udsat for:

2.2 Hvem har udsat dig for diskrimination? (Sæt kryds i en eller flere)

❑ 2.2.1 Andre studerende
❑ 2.2.2 Underviser/lærer
❑ 2.2.3 Læge
❑ 2.2.4 Sygeplejerske eller andet sundhedspersonale
❑ 2.2.5 Ledelse i sundhedssektoren
❑ 2.2.6 Administrativt personale i sundhedssektoren
❑ 2.2.7 Administrativt personale på universitetet
❑ 2.2.8 Patienter
❑ 2.2.9 Pårørende til patienter
❑ 2.2.10 Andre__________

2.3 Hvad tror du er baggrunden for dine oplevelser? (Sæt kryds i en eller flere)

❑ 2.3.1 Mit køn eller kønsidentitet
❑ 2.3.2 Min etnicitet
❑ 2.3.3 Min hudfarve
❑ 2.3.4 Mit sprog
❑ 2.3.5 Min seksuelle orientering
❑ 2.3.6 Min religion

- ❏ 2.3.7 Min alder
- ❏ 2.3.8 Min funktionsnedsættelse
- ❏ 2.3.9 Andet__________

2.4 Har du noget du vil tilføje eller vil du beskrive dine oplevelser, så kan du gøre det her:

3. Spørgsmål om din baggrund

3.1 Hvor er du indskrevet?

- ❏ Klinisk basisuddannelse
- ❏ Aalborg Universitet
- ❏ Aarhus Universitet
- ❏ Københavns Universitet
- ❏ Syddansk Universitet

3.2 Hvilket semester på studiet er du på?

- ❏ 7. semester
- ❏ 8. semester
- ❏ 9. semester
- ❏ 10. semester
- ❏ 11. semester
- ❏ 12. semester

3.3 Hvilket køn identificere du dig som?

- ❏ Kvinde
- ❏ Mand
- ❏ Non-binær
- ❏ Fortrækker ikke at oplyse det
- ❏ Fortrækker at beskrive det selv __________

3.4 Hvilken seksuel orientering repræsenterer dig bedst?

- ❏ Heteroseksuel
- ❏ Homoseksuel mand
- ❏ Homoseksuel kvinde
- ❏ Biseksuel
- ❏ Foretrækker ikke at oplyse det
- ❏ Foretrækker at beskrive det selv __________

3.5 Hvilket år er du født?

____

3.6 I hvilket land er du født?

____

3.7 Opfatter du dig som værende en del af en etnisk minoritet i det land du er bosat i?

- ☐ Ja
- ☐ Nej

3.7.a Beskriv venligst din etnicitet: f.eks. dansk-tyrkisk, tysk, dansk-palæstinensisk, somalisk, grønlandsk

____

3.8 I hvilket land er dine forældre født?

Forælder 1 __________

Forælder 2 __________

3.9 Hvad er den højeste uddannelse gennemført af en af dine forældre?

- ☐ Folkeskole til 9. eller 10. klasse
- ☐ Et eller flere kortere kurser (fx specialarbejderkurser, arbejdsmarkedskurser m.v.)
- ☐ Gymnasial uddannelser (fx studenterexamen, HF, HHX, HTX)
- ☐ Erhvervsfaglig uddannelse/faglært (fx kontor- eller butiksassistent, frisør, murer, lægesekretær, social- og sundhedshjælper/assistent, landmand)
- ☐ Kort videregående uddannelse, 2-3 år (fx markedsøkonom, politibetjent, laborant, maskintekniker, datamatiker, multimediedesigner, økonoma, tandplejer)
- ☐ Mellemlang videregående uddannelse, 3-4 år (fx folkeskolelærer, socialrådgiver, bygningskonstruktør, sygeplejerske, fysioterapeut, diplomingeniør, pædagog, bachelor)
- ☐ Lang videregående uddannelse, mere end 4 år (fx civilingeniør, cand.mag., læge, psykolog)
- ☐ Anden uddannelse: __________

3.10 Har du en funktionsnedsættelse?

- ☐ Ja
- ☐ Nej

3.10. a Beskriv din funktionsnedsættelse

Må vi kontakte dig i forbindelse med en opfølgende kvalitativ undersøgelse? Hvis ja, skriv din e-mail her:

Husk at trykke "afslut".

Tak for din besvarelse!
