# Supplementary material for: Do Danish medical students feel prepared to deliver healthcare to patients with backgrounds different from their own? A cross-sectional survey
Source: BMC Med Educ. 2024 Nov 26;24:1366. doi: 10.1186/s12909-024-06371-5 (PMC11590545; doi:10.1186/s12909-024-06371-5)
Supplement: Supplementary file 2 — Supplementary Material 2 [file 12909_2024_6371_MOESM2_ESM.docx]

**Appendix 2 - Characteristics of the informants**

|  | **N (645)** | | **%** |
| --- | --- | --- | --- |
| **Enrolled at University or Clinical Basic Education** | | | |
|  | | | |
| Aarhus University | 199 | 30,9 | |
| University of Copenhagen | 222 | 34.4 | |
| University of Southern Denmark | 100 | 15.5 | |
| Aalborg University | 36 | 5.6 | |
| Clinical Basic Education | 88 | 13.6 | |
|  |  |  | |
| **Semester, if enrolled at University (N =557)** |  |  | |
| 7. semester | 52 | 9.3 | |
| 8. semester | 84 | 15.1 | |
| 9. semester | 82 | 14.7 | |
| 10. semester | 97 | 17.4 | |
| 11. semester | 82 | 14.7 | |
| 12. semester | 160 | 28.7 | |
|  |  |  | |
| **Year of birth** |  |  | |
| 1970-1989 | 15 | 2.6 | |
| 1990-1999 | 621 | 96.2 | |
| 2000- | 9 | 1.4 | |
|  |  |  | |
| **Gender** |  |  | |
| Female | 453 | 70.2 | |
| Male | 184 | 28.5 | |
| Non-binary/self-identified | 7 | 1.1 | |
| Prefer not to disclose | 1 | 0.2 | |
|  |  |  | |
| **Sexual orientation** |  |  | |
| Heterosexual | 565 | 87.6 | |
| Homosexual man | 17 | 2.6 | |
| Homosexual woman | 9 | 1.4 | |
| Bisexual | 34 | 5.3 | |
| Self-identified | 10 | 1.6 | |
| Prefer not to disclose | 10 | 1.6 | |
|  |  |  | |
| **Self- identified ethnicity (grouped)** |  |  | |
| Danish/European | 580 | 89.9 | |
| African/Middle Eastern/South American/Asian | 58 | 9.0 | |
| Prefer not to disclose/Other | 7 | 1.1 | |
|  |  |  | |
| (**Dis)Abilities** |  |  | |
| Yes | 43 | 6.7 | |
| No | 602 | 93.3 | |
|  |  |  | |
| **Parents’ highest education** |  |  | |
| Elementary School | 10 | 1.6 | |
| One or more shorter courses | 8 | 1.2 | |
| High School or equivalent | 19 | 2.9 | |
| Vocational education/skilled | 81 | 12.6 | |
| Short higher education, 2-3 years | 49 | 7.6 | |
| Middel long higher education, 3-4 years | 157 | 24.3 | |
| Long higher education, more than 4 years | 317 | 49.1 | |
| Other education eg. PhD | 4 | 0.6 | |
