# Supplementary material for: Do Danish medical students feel prepared to deliver healthcare to patients with backgrounds different from their own? A cross-sectional survey
Source: BMC Med Educ. 2024 Nov 26;24:1366. doi: 10.1186/s12909-024-06371-5 (PMC11590545; doi:10.1186/s12909-024-06371-5)
Supplement: Supplementary file 3 — Supplementary Material 3 [file 12909_2024_6371_MOESM3_ESM.docx]

| How Prepared Do You Feel to Care for Patients.. |  | **UCPH**  7-9. semester | **AAU**  10-12. semester | **AU**  10-12. semester | **UCPH**  10-12. semester | **SDU**  10-12. semester | **CBE** | **AAU**  7-9. semester | **AU**  7-9. semester | **SDU**  7-9. semester |
| --- | --- | --- | --- | --- | --- | --- | --- | --- | --- | --- |
| 1.1.11 … from cultures different from your own? | OR | Ref | 0.41 | 0.5 | 0.74 | 0.5 | 0.9 | 0.32 | 1.08 | 0.47 |
|  | CI 95% | Ref | [0.15;1.15] | [0.28;0.89] | [0.42;1.29] | [0.25;0.99] | [0.49;1.63] | [0.08;1.27] | [0.58;2.02] | [0.19;1.14] |
|  | p-value | Ref | 0.09 | 0.019 | 0.29 | 0.048 | 0.72 | 0.1 | 0.81 | 0.096 |
| 1.1.12 … with health beliefs or practices at odds with Western medicine? | OR | Ref | 0.32 | 0.45 | 0.71 | 0.4 | 0.87 | 0.95 | 0.93 | 0.52 |
|  | CI 95% | Ref | [0.12;0.84] | [0.25;0.81] | [0.39;1.29] | [0.20;0.80] | [0.45;1.69] | [0.26;3.46] | [0.46;1.86] | [0.22;1.24] |
|  | p-value | Ref | 0.02 | 0.008 | 0.26 | 0.0094 | 0.69 | 0.94 | 0.83 | 0.14 |
| 1.1.13 … with a distrust of the Danish healthcare system? | OR | Ref | 0.63 | 0.62 | 1.08 | 0.54 | 1.51 | 0.91 | 0.79 | 0.38 |
|  | CI 95% | Ref | [0.23;1.70] | [0.33;1.14] | [0.57;2.02] | [0.27;1.11] | [0.74;3.10] | [0.24;3.37] | [0.39;1.60] | [0.16;0.92] |
|  | p-value | Ref | 0.36 | 0.12 | 0.82 | 0.093 | 0.26 | 0.88 | 0.51 | 0.031 |
| 1.1.14 … with limited Danish language proficiency? | OR | Ref | 0.35 | 0.75 | 1.38 | 0.72 | 0.86 | 0.59 | 1.11 | 0.71 |
|  | CI 95% | Ref | [0.12;0.97] | [0.43;1.30] | [0.79;2.39] | [0.37;1.39] | [0.47;1.56] | [0.18;1.98] | [0.60;2.08] | [0.30;1.65] |
|  | p-value | Ref | 0.044 | 0.31 | 0.26 | 0.32 | 0.62 | 0.39 | 0.74 | 0.42 |
| 1.1.15 … who are newly arrived immigrants? | OR | Ref | 0.51 | 0.68 | 1.51 | 0.51 | 1.12 | 0.69 | 0.98 | 0.5 |
|  | CI 95% | Ref | [0.19;1.41] | [0.36;1.30] | [0.77;2.98] | [0.24;1.06] | [0.54;2.32] | [0.18;2.60] | [0.46;2.08] | [0.20;1.25] |
|  | p-value | Ref | 0.2 | 0.24 | 0.23 | 0.073 | 0.75 | 0.58 | 0.96 | 0.14 |
| 1.1.16 … whose religious beliefs affect treatment? | OR | Ref | 0.35 | 0.49 | 0.75 | 0.37 | 0.6 | 0.21 | 0.88 | 0.31 |
|  | CI 95% | Ref | [0.13;0.95] | [0.25;0.94] | [0.38;1.44] | [0.17;0.78] | [0.29;1.22] | [0.06;0.73] | [0.41;1.91] | [0.12;0.78] |
|  | p-value | Ref | 0.04 | 0.031 | 0.38 | 0.009 | 0.16 | 0.014 | 0.75 | 0.013 |
| 1.1.17… who use alternative medicine? | OR | Ref | 0.56 | 0.68 | 1.68 | 0.7 | 0.82 | 0.83 | 0.95 | 0.69 |
|  | CI 95% | Ref | [0.21;1.47] | [0.39;1.20] | [0.95;2.94] | [0.36;1.37] | [0.45;1.50] | [0.25;2.75] | [0.51;1.78] | [0.29;1.61] |
|  | p-value | Ref | 0.24 | 0.18 | 0.072 | 0.3 | 0.52 | 0.76 | 0.87 | 0.39 |
| 1.1.18 … who are members of ethnic minorities? | OR | Ref | 0.67 | 0.48 | 0.72 | 0.51 | 0.83 | 0.1 | 1.39 | 0.57 |
|  | CI 95% | Ref | [0.25;1.79] | [0.27;0.87] | [0.41;1.27] | [0.25;1.02] | [0.45;1.52] | [0.01;0.79] | [0.74;2.61] | [0.23;1.37] |
|  | p-value | Ref | 0.42 | 0.015 | 0.26 | 0.057 | 0.54 | 0.029 | 0.31 | 0.21 |
| Notes: Multiple logistic regression model adjusted for gender, ethnicity and parents’ education, and an interaction between university and semester | | | | | | | | | | |

**Appendix C – Tables showing preparedness across Universities, Semesters and Clinical Basic Education (CBE) and sensitivity analyses**

Tabel 5 - The importance of Universities, Semester and Clinical Basic Education (CBE)

for medical students’ preparedness to care for patients of backgrounds or characteristics different from their own

Table 6. The importance of semesters for medical students’ feeling of being helpless in relation to the treatment of patients of a culture different from their own

| **Question** | **University** | **Semester** | **Odds ratio** | **95% CI** | **p-value** |
| --- | --- | --- | --- | --- | --- |
| 1.1.19…In the last year, how often have you felt helpless about what to do when providing care involving patients of a culture different from your own? | **UCPH** | 7-9. semester | Ref | - | - |
|  | **AAU** | 10-12. semester | 0.75 | [0.26;2.15] | 0.59 |
|  | **AU** | 10-12. semester | 0.84 | [0.44;1.60] | 0.6 |
|  | **UCPH** | 10-12. semester | 0.97 | [0.53;1.81] | 0.94 |
|  | **SDU** | 10-12. semester | 0.91 | [0.43;1.92] | 0.81 |
|  | **CBE** |  | 1.39 | [0.70;2.75] | 0.34 |
|  | **AAU** | 7-9. semester | 0.8 | [0.20;3.21] | 0.76 |
|  | **AU** | 7-9. semester | 1.5 | [0.72;3.14] | 0.28 |
|  | **SDU** | 7-9. semester | 0.61 | [0.21;1.76] | 0.36 |

Notes: Multiple logistic regression model adjusted for gender, ethnicity and parents’ education, and an interaction between university and semester

Table 7. Sensitivity analysis (multiple logistic regression with semester and gender separate without clinical basic education)

| **Question** | **Variabel** | **Unit** | **OR** | **CI 95%** | **p-value** |
| --- | --- | --- | --- | --- | --- |
| How Prepared Do You Feel to Care for Patients.. |  |  |  |  |  |
| 1.1.11 … from cultures different from your own? | University | UCPH | Ref |  | 0.099 |
|  |  | AAU | 0.45 | [0.20;1.01] | 0.054 |
|  |  | AU | 0.83 | [0.55;1.25] | 0.38 |
|  |  | SDU | 0.6 | [0.35;1.03] | 0.063 |
|  | Semester | 7-9. semester | Ref |  | 0.037 |
|  |  | 10-12. semester | 0.68 | [0.47;0.98] | 0.037 |
|  | Gender | Women | Ref |  | 0.00066 |
|  |  | Men | 0.5 | [0.33;0.75] | 0.00089 |
|  | Parents Education | Long | Ref |  | 0.61 |
|  |  | Secondary education | 0.54 | [0.25;1.18] | 0.12 |
|  |  | Vocational education/trade worked | 1.02 | [0.57;1.83] | 0.95 |
|  |  | Short | 1.04 | [0.52;2.06] | 0.91 |
|  |  | Medium | 0.98 | [0.63;1.53] | 0.94 |
|  | Ethnicity | Danish/ European | Ref |  | 0.86 |
|  |  | African/Middle Eastern/ South American/Asian | 1.06 | [0.58;1.93] | 0.86 |
| 1.1.12 …with health beliefs or practices at odds with Western medicine? | University | UCPH | Ref |  | 0.11 |
|  |  | AAU | 0.57 | [0.27;1.20] | 0.14 |
|  |  | AU | 0.73 | [0.48;1.12] | 0.15 |
|  |  | SDU | 0.56 | [0.33;0.94] | 0.027 |
|  | Semester | 7-9. semester | Ref |  | 0.0056 |
|  |  | 10-12. semester | 0.59 | [0.41;0.86] | 0.0061 |
|  | Gender | Women | Ref |  | 0.00022 |
|  |  | Men | 0.48 | [0.33;0.71] | 0.00023 |
|  | Parents Education | Long | Ref |  | 0.51 |
|  |  | Secondary education | 0.77 | [0.37;1.58] | 0.48 |
|  |  | Vocational education/trade worked | 0.86 | [0.48;1.55] | 0.62 |
|  |  | Short | 0.56 | [0.29;1.10] | 0.092 |
|  |  | Medium | 0.8 | [0.52;1.26] | 0.34 |
|  | Ethnicity | Danish/ European | Ref |  | 0.41 |
|  |  | African/Middle Eastern/ South American/Asian | 0.77 | [0.42;1.42] | 0.41 |
| 1.1.13 …with a distrust of the Danish healthcare system? | University | UCPH | Ref |  | 0.053 |
|  |  | AAU | 0.68 | [0.31;1.48] | 0.34 |
|  |  | AU | 0.66 | [0.42;1.03] | 0.069 |
|  |  | SDU | 0.48 | [0.28;0.83] | 0.0078 |
|  | Semester | 7-9. semester | Ref |  | 0.89 |
|  |  | 10-12. semester | 0.97 | [0.66;1.43] | 0.89 |
|  | Gender | Women | Ref |  | <0.0001 |
|  |  | Men | 0.33 | [0.22;0.49] | <0.0001 |
|  | Parents Education | Long | Ref |  | 0.9 |
|  |  | Secondary education | 1.14 | [0.52;2.50] | 0.75 |
|  |  | Vocational education/trade worked | 0.94 | [0.51;1.73] | 0.83 |
|  |  | Short | 0.77 | [0.39;1.52] | 0.45 |
|  |  | Medium | 0.86 | [0.54;1.36] | 0.52 |
|  | Ethnicity | Danish/ European | Ref |  | 0.68 |
|  |  | African/Middle Eastern/ South American/Asian | 1.15 | [0.59;2.24] | 0.68 |
| 1.1.14 …with limited Danish proficiency? | University | UCPH | Ref |  | 0.02 |
|  |  | AAU | 0.35 | [0.16;0.76] | 0.0078 |
|  |  | AU | 0.73 | [0.49;1.09] | 0.13 |
|  |  | SDU | 0.59 | [0.36;0.98] | 0.043 |
|  | Semester | 7-9. semester | Ref |  | 0.81 |
|  |  | 10-12. semester | 0.96 | [0.67;1.37] | 0.81 |
|  | Gender | Women | Ref |  | 0.00073 |
|  |  | Men | 0.52 | [0.35;0.76] | 0.00084 |
|  | Parents Education | Long | Ref |  | 0.86 |
|  |  | Secondary education | 0.95 | [0.47;1.93] | 0.89 |
|  |  | Vocational education/trade worked | 0.95 | [0.54;1.69] | 0.86 |
|  |  | Short | 0.92 | [0.48;1.79] | 0.82 |
|  |  | Medium | 0.78 | [0.51;1.20] | 0.25 |
|  | Ethnicity | Danish/ European | Ref |  | 0.98 |
|  |  | African/Middle Eastern/ South American/Asian | 1.01 | [0.56;1.81] | 0.98 |
| 1.1.15 …who are newly arrived immigrants? | University | UCPH | Ref |  | 0.012 |
|  |  | AAU | 0.45 | [0.21;0.99] | 0.048 |
|  |  | AU | 0.63 | [0.39;1.02] | 0.06 |
|  |  | SDU | 0.42 | [0.24;0.73] | 0.0022 |
|  | Semester | 7-9. semester | Ref |  | 0.97 |
|  |  | 10-12. semester | 0.99 | [0.66;1.49] | 0.97 |
|  | Gender | Women | Ref |  | <0.0001 |
|  |  | Men | 0.34 | [0.23;0.52] | <0.0001 |
|  | Parents Education | Long | Ref |  | 0.43 |
|  |  | Secondary education | 0.49 | [0.23;1.04] | 0.062 |
|  |  | Vocational education/trade worked | 1.02 | [0.53;1.97] | 0.94 |
|  |  | Short | 1.13 | [0.54;2.40] | 0.74 |
|  |  | Medium | 0.93 | [0.57;1.53] | 0.79 |
|  | Ethnicity | Danish/ European | Ref |  | 0.41 |
|  |  | African/Middle Eastern/ South American/Asian | 0.75 | [0.39;1.46] | 0.4 |
| 1.1.16 … whose religious beliefs affect treatment? | University | UCPH | Ref |  | 0.0038 |
|  |  | AAU | 0.35 | [0.16;0.74] | 0.0063 |
|  |  | AU | 0.72 | [0.46;1.15] | 0.17 |
|  |  | SDU | 0.43 | [0.25;0.74] | 0.0023 |
|  | Semester | 7-9. semester | Ref |  | 0.23 |
|  |  | 10-12. semester | 0.78 | [0.52;1.17] | 0.23 |
|  | Gender | Women | Ref |  | <0.0001 |
|  |  | Men | 0.29 | [0.20;0.44] | <0.0001 |
|  | Parents Education | Long | Ref |  | 0.96 |
|  |  | Secondary education | 1.03 | [0.47;2.25] | 0.95 |
|  |  | Vocational education/trade worked | 1.08 | [0.57;2.06] | 0.81 |
|  |  | Short | 0.91 | [0.45;1.84] | 0.79 |
|  |  | Medium | 0.86 | [0.53;1.38] | 0.53 |
|  | Ethnicity | Danish/ European | Ref |  | 0.19 |
|  |  | African/Middle Eastern/ South American/Asian | 0.65 | [0.34;1.23] | 0.18 |
| 1.1.17 …who use alternative medicines? | University | UCPH | Ref |  | 0.016 |
|  |  | AAU | 0.48 | [0.23;1.01] | 0.054 |
|  |  | AU | 0.59 | [0.39;0.88] | 0.01 |
|  |  | SDU | 0.53 | [0.31;0.88] | 0.015 |
|  | Semester | 7-9. semester | Ref |  | 0.7 |
|  |  | 10-12. semester | 1.07 | [0.75;1.54] | 0.7 |
|  | Gender | Women | Ref |  | <0.0001 |
|  |  | Men | 0.43 | [0.29;0.64] | <0.0001 |
|  | Parents Education | Long | Ref |  | 0.89 |
|  |  | Secondary education | 0.83 | [0.40;1.70] | 0.6 |
|  |  | Vocational education/trade worked | 0.92 | [0.52;1.65] | 0.79 |
|  |  | Short | 1.03 | [0.53;2.01] | 0.92 |
|  |  | Medium | 0.81 | [0.52;1.25] | 0.34 |
|  | Ethnicity | Danish/ European | Ref |  | 0.018 |
|  |  | African/Middle Eastern/ South American/Asian | 2.1 | [1.12;3.92] | 0.02 |
| 1.1.18 …who are members of ethnic minorities? | University | UCPH | Ref |  | 0.2 |
|  |  | AAU | 0.5 | [0.22;1.13] | 0.095 |
|  |  | AU | 0.92 | [0.61;1.38] | 0.68 |
|  |  | SDU | 0.66 | [0.39;1.13] | 0.13 |
|  | Semester | 7-9. semester | Ref |  | 0.014 |
|  |  | 10-12. semester | 0.63 | [0.44;0.91] | 0.013 |
|  | Gender | Women | Ref |  | 0.00058 |
|  |  | Men | 0.49 | [0.32;0.74] | 0.00079 |
|  | Parents Education | Long | Ref |  | 0.76 |
|  |  | Secondary education | 0.7 | [0.33;1.49] | 0.36 |
|  |  | Vocational education/trade worked | 0.76 | [0.41;1.39] | 0.37 |
|  |  | Short | 1.06 | [0.53;2.10] | 0.87 |
|  |  | Medium | 1.04 | [0.67;1.61] | 0.88 |
|  | Ethnicity | Danish/ European | Ref |  | 0.38 |
|  |  | African/Middle Eastern/ South American/Asian | 1.3 | [0.72;2.37] | 0.38 |
| 1.1.19…In the last year, how often have you felt helpless about what to do when providing care involving patients of a culture different from your own? | University | UCPH | 0.79 | [0.35;1.79] | 0.56 |
|  |  | AAU | 1.07 | [0.67;1.69] | 0.79 |
|  |  | AU | 0.86 | [0.48;1.53] | 0.6 |
|  |  | SDU | Ref |  | 0.45 |
|  | Semester | 7-9. semester | 0.85 | [0.57;1.29] | 0.45 |
|  |  | 10-12. semester | Ref |  | 0.00031 |
|  | Gender | Women | 0.44 | [0.28;0.70] | 0.00043 |
|  |  | Men | Ref |  | 0.0065 |
|  | Parents Education | Long | 0.28 | [0.12;0.69] | 0.0058 |
|  |  | Secondary education | 0.38 | [0.19;0.77] | 0.0072 |
|  |  | Vocational education/trade worked | 0.86 | [0.41;1.82] | 0.7 |
|  |  | Short | 0.72 | [0.44;1.18] | 0.19 |
|  |  | Medium | Ref |  | 0.013 |
|  | Ethnicity | Danish/ European | 0.79 | [0.35;1.79] | 0.56 |
|  |  | African/Middle Eastern/ South American/Asian | 1.07 | [0.67;1.69] | 0.79 |

Table 8. Sensitivity analysis (multiple logistic regression with semester and gender separate for clinical basic education)

| **Question** | **Variabel** | **Unit** | **OR** | **CI 95%** | **p-value** |
| --- | --- | --- | --- | --- | --- |
| How Prepared Do You Feel to Care for Patients.. |  |  |  |  |  |
| 1.1.11 … from cultures different from your own? | Gender | Women | Ref |  | 0.012 |
|  |  | Men | 0.26 | [0.09;0.78] | 0.016 |
|  | Parents Education | Long | Ref |  | 0.38 |
|  |  | Secondary education | 0.42 | [0.03;5.50] | 0.51 |
|  |  | Vocational education/trade worked | 0.96 | [0.28;3.27] | 0.95 |
|  |  | Short | 0.22 | [0.02;2.17] | 0.19 |
|  |  | Medium | 1.71 | [0.56;5.29] | 0.35 |
|  | Ethnicity | Danish/ European | Ref |  | 0.94 |
|  |  | African/Middle Eastern/ South American/Asian | 1.08 | [0.13;9.19] | 0.94 |
| 1.1.12 … with health beliefs or practices at odds with Western medicine? | Gender | Women | Ref |  | 0.0041 |
|  |  | Men | 0.2 | [0.06;0.62] | 0.0055 |
|  | Parents Education | Long | Ref |  | 0.061 |
|  |  | Secondary education | 0.26 | [0.01;4.76] | 0.36 |
|  |  | Vocational education/trade worked | 0.36 | [0.09;1.37] | 0.13 |
|  |  | Short | 0.39 | [0.05;2.86] | 0.35 |
|  |  | Medium | 3.91 | [0.75;20.45] | 0.11 |
|  | Ethnicity | Danish/ European | Ref |  | 0.085 |
|  |  | African/Middle Eastern/ South American/Asian | Inf | [0.00;Inf] | 0.99 |
| 1.1.13 … with a distrust of the Danish healthcare system? | Gender | Women | Ref |  | 0.02 |
|  |  | Men | 0.24 | [0.07;0.82] | 0.023 |
|  | Parents Education | Long | Ref |  | 0.024 |
|  |  | Secondary education | Inf | [0.00;Inf] | 1 |
|  |  | Vocational education/trade worked | 1.39 | [0.31;6.24] | 0.67 |
|  |  | Short | 1.02 | [0.10;10.96] | 0.99 |
|  |  | Medium | Inf | [0.00;Inf] | 0.99 |
|  | Ethnicity | Danish/ European | Ref |  | 0.27 |
|  |  | African/Middle Eastern/ South American/Asian | Inf | [0.00;Inf] | 1 |
| 1.1.14 … with limited Danish language proficiency? | Gender | Women | Ref |  | 0.026 |
|  |  | Men | 0.31 | [0.11;0.90] | 0.031 |
|  | Parents Education | Long | Ref |  | 0.6 |
|  |  | Secondary education | 0.21 | [0.01;3.49] | 0.28 |
|  |  | Vocational education/trade worked | 0.53 | [0.15;1.87] | 0.32 |
|  |  | Short | 0.48 | [0.07;3.29] | 0.45 |
|  |  | Medium | 1.1 | [0.36;3.35] | 0.86 |
|  | Ethnicity | Danish/ European | Ref |  | 0.25 |
|  |  | African/Middle Eastern/ South American/Asian | 4.09 | [0.30;55.51] | 0.29 |
| 1.1.15 … who are newly arrived immigrants? | Gender | Women | Ref |  | <0.0001 |
|  |  | Men | 0.06 | [0.01;0.25] | 0.00011 |
|  | Parents Education | Long | Ref |  | 0.22 |
|  |  | Secondary education | 0.17 | [0.01;2.96] | 0.23 |
|  |  | Vocational education/trade worked | 0.27 | [0.05;1.40] | 0.12 |
|  |  | Short | Inf | [0.00;Inf] | 0.99 |
|  |  | Medium | 1.99 | [0.32;12.30] | 0.46 |
|  | Ethnicity | Danish/ European | Ref |  | 0.64 |
|  |  | African/Middle Eastern/ South American/Asian | 0.51 | [0.04;7.48] | 0.63 |
| 1.1.16 … whose religious beliefs affect treatment? | Gender | Women | Ref |  | <0.0001 |
|  |  | Men | 0.09 | [0.03;0.30] | <0.0001 |
|  | Parents Education | Long | Ref |  | 0.55 |
|  |  | Secondary education | 0.27 | [0.02;4.06] | 0.34 |
|  |  | Vocational education/trade worked | 0.3 | [0.07;1.31] | 0.11 |
|  |  | Short | 0.49 | [0.04;5.58] | 0.57 |
|  |  | Medium | 0.71 | [0.17;2.94] | 0.64 |
|  | Ethnicity | Danish/ European | Ref |  | 0.84 |
|  |  | African/Middle Eastern/ South American/Asian | 0.76 | [0.06;9.99] | 0.84 |
| 1.1.17… who use alternative medicine? | Gender | Women | Ref |  | 0.13 |
|  |  | Men | 0.45 | [0.16;1.28] | 0.13 |
|  | Parents Education | Long | Ref |  | 0.73 |
|  |  | Secondary education | 1.93 | [0.14;25.78] | 0.62 |
|  |  | Vocational education/trade worked | 2.18 | [0.64;7.46] | 0.21 |
|  |  | Short | 0.87 | [0.13;5.97] | 0.89 |
|  |  | Medium | 1.52 | [0.51;4.57] | 0.45 |
|  | Ethnicity | Danish/ European | Ref |  | 0.37 |
|  |  | African/Middle Eastern/ South American/Asian | 2.82 | [0.25;31.40] | 0.4 |
| 1.1.18 … who are members of ethnic minorities? | Gender | Women | Ref |  | <0.0001 |
|  |  | Men | 0.03 | [0.00;0.23] | 0.00088 |
|  | Parents Education | Long | Ref |  | 0.29 |
|  |  | Secondary education | 0.3 | [0.02;4.00] | 0.36 |
|  |  | Vocational education/trade worked | 0.31 | [0.07;1.25] | 0.099 |
|  |  | Short | 0.42 | [0.06;2.93] | 0.38 |
|  |  | Medium | 1.3 | [0.37;4.56] | 0.69 |
|  | Ethnicity | Danish/ European | Ref |  | 0.91 |
|  |  | African/Middle Eastern/ South American/Asian | 1.14 | [0.13;10.39] | 0.91 |
| 1.1.19…In the last year, how often have you felt helpless about what to do when providing care involving patients of a culture different from your own? | Gender | Women | Ref |  | 0.098 |
|  |  | Men | 0.39 | [0.12;1.22] | 0.11 |
|  | Parents Education | Long | Ref |  | 0.94 |
|  |  | Secondary education | 0.93 | [0.05;16.63] | 0.96 |
|  |  | Vocational education/trade worked | 0.83 | [0.21;3.24] | 0.78 |
|  |  | Short | 1.86 | [0.15;23.33] | 0.63 |
|  |  | Medium | 1.43 | [0.42;4.91] | 0.57 |
|  | Ethnicity | Danish/ European | Ref |  | 0.06 |
|  |  | African/Middle Eastern/ South American/Asian | Inf | [0.00;Inf] | 0.99 |
